# Severe Mitral Valve Regurgitation in Infective Endocarditis: A Case Report

**DOI:** 10.7759/cureus.60515

**Published:** 2024-05-17

**Authors:** Alexandra Paula, Denise Pinto, Maria José Duarte, José Vaz

**Affiliations:** 1 Intensive Care Unit, Hospital José Joaquim Fernandes, Beja, PRT; 2 Cardiology, Hospital José Joaquim Fernandes, Beja, PRT

**Keywords:** streptococcus mitis bacteremia, mitral regurgitation, lung injury after cardiopulmonary bypass, infective endocarditis, bicuspid aortic valve disease

## Abstract

We present a case report of a patient with infective endocarditis. He came to the emergency room with respiratory failure due to severe pneumonia and pulmonary edema. On 2D transesophageal echocardiography, vegetations were seen in both mitral and aortic valves, with mitral valve perforation and severe regurgitation. His clinical presentation and severity of the disease made him suitable for urgent valve repair. He was submitted to mitral valvuloplasty with closure of the valve perforation and insertion of a bioprosthetic aortic valve. Despite significant clinical improvement, a post-surgical complication was noted with new-onset lung injury after cardiopulmonary bypass. This is an interesting case of a patient with suspected retrograde valve involvement, affecting the aortic valve, the mitral-aortic intervalvular fibrosa, and the mitral valve, ending with mitral valve abscess with leaflet perforation and valvular regurgitation.

## Introduction

Infective endocarditis is a disease with a rising incidence. In 2019, it had an age-standardized incident rate of 13.80% in comparison with 9.91% in 1990. Its 25% mortality makes it extremely important to rapidly diagnose and treat [1]. Its diagnosis is based on Duke's criteria, recently updated in 2023. It includes pathologic and clinical criteria, divided into microbiological, imaging, and surgical evidence. According to the presence of major and minor diagnostic criteria, a patient can have a definitive, possible, or rejected diagnosis [2].

Surgical criteria have recently been included due to the importance of direct visualization of the valve, even if there is no following microbiological confirmation of infection. Surgical valve repair is necessary for a significant number of patients where heart failure is present (the percentage varies depending on the study performed but Revilla et al. have reported a 72% incidence) [3,4]. However, a surgical approach is not risk-exempt, and potential complications should be considered regarding it and its timing. The surgical approach is performed under extracorporeal circulation, which has some possible adverse effects that should be taken into consideration in a patient who is sick enough to need surgical valve repair.

Our case report illustrates a severe case of cardiogenic and septic shock due to infective endocarditis and pneumonia. Despite an appropriate medical approach, this patient persisted in respiratory failure, needing urgent surgical treatment. After valve repair, the presence of new-onset lung injury put on the hypothesis of an intra-operatory complication.

## Case presentation

A 53-year-old male patient with no known past medical history other than poor dentition was admitted to the emergency room of a secondary care hospital with a 24-hour history of shortness of breath. He reported a fever for two weeks, a productive cough, and mucopurulent sputum. He was brought in by the pre-hospital medical emergency team under continuous positive airway pressure with FiO_2_ 100%. He was diaphoretic and had signs of respiratory distress; on auscultation, bronchospasm and diffuse crackles were found, and a grade 4/6 systolic murmur was noted. SpO_2_ was 80%.

**Figure 1:**
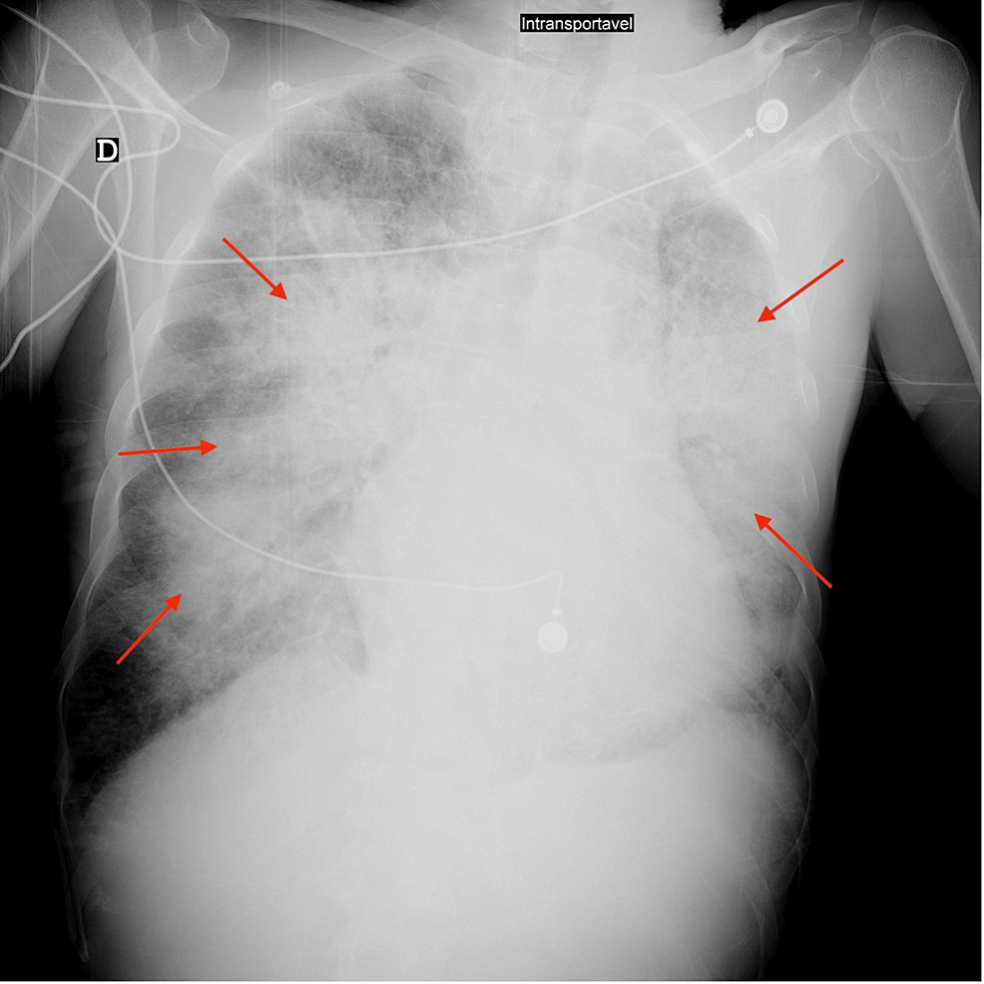
X-ray performed showing bilateral infiltrates AP view X-ray shows bilateral infiltrates (arrows)

**Figure 2:**
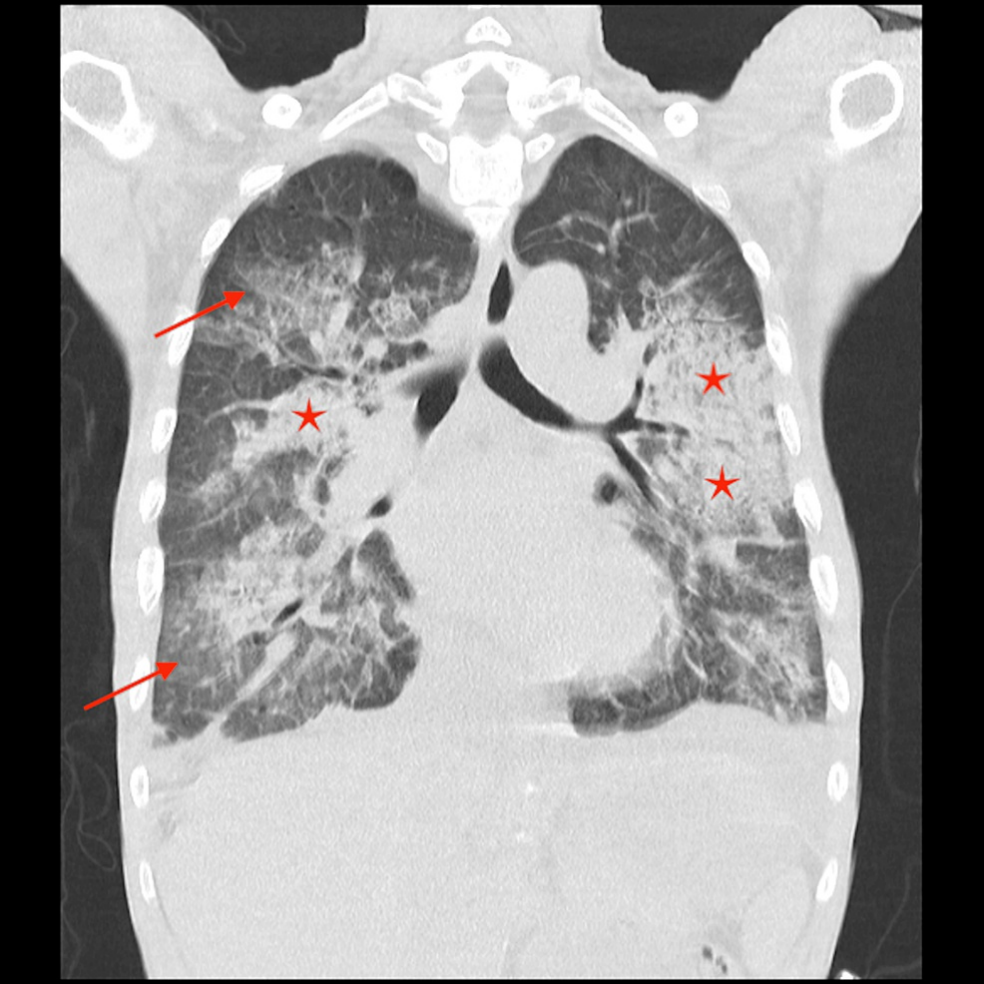
Chest CT scan showing bilateral pneumonia Bilateral and diffuse alveolar opacities (arrows) and multifocal areas of airspace consolidation (stars) with preserved lung volumes compatible with bilateral pneumonia. CT, computed tomography

Lab results showed a pO_2_ 39 mmHg with a normal pCO_2_ and lactate elevation of 3.5 mmoL/L. Chest X-ray (Figure 1) and chest computed tomography (CT) scan (Figure 2) showed bilateral pneumonia. Cultures were collected and Streptococcus spp was identified on a multiplex test from blood and respiratory specimens.

**Figure 3:**
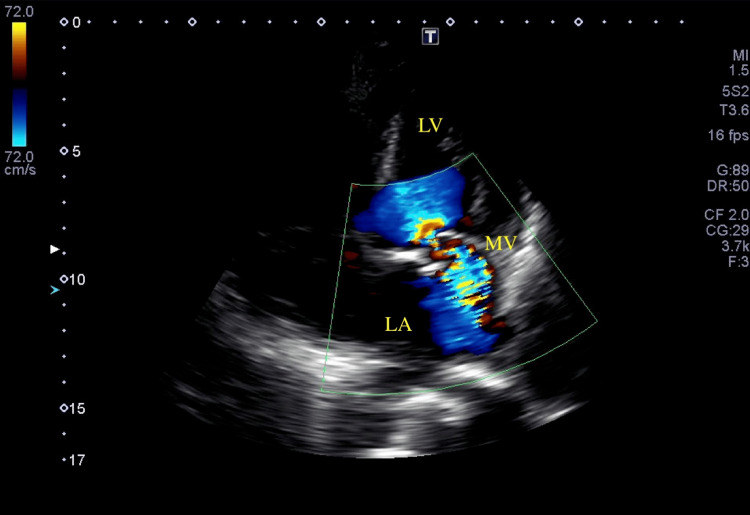
Transthoracic echocardiography in apical four-chamber view showing severe mitral regurgitation LV, left ventricle; LA, left atrium; MV, mitral valve

**Figure 4:**
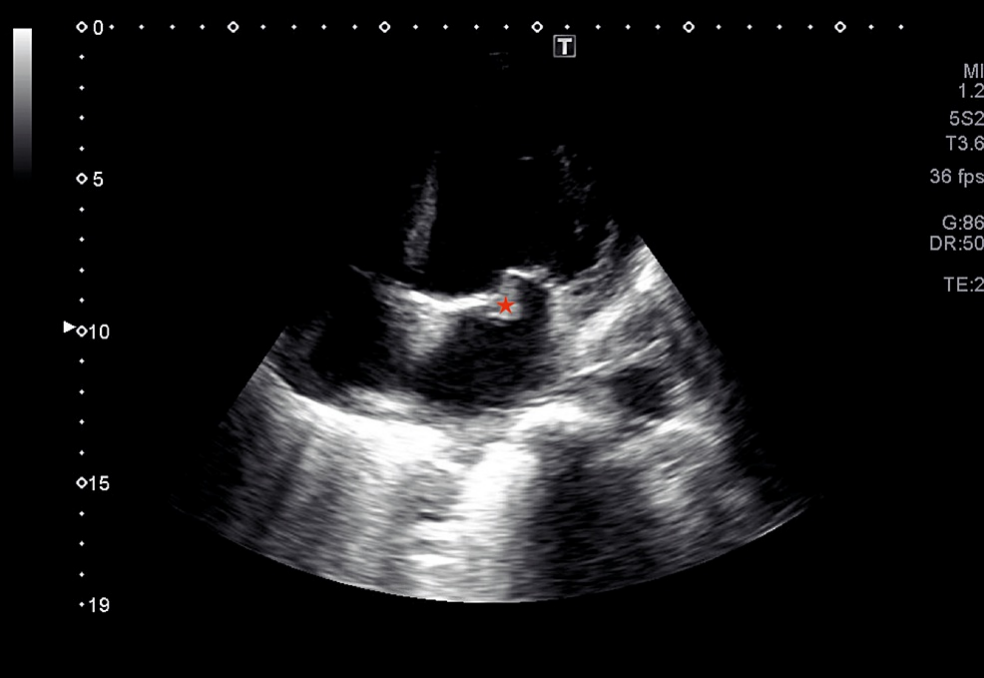
Transthoracic echocardiography showing mitral valve Mitral valve leaflet thickening and calcification can be seen (star) in apical four-chamber view echocardiography.

He was admitted to the intensive care unit under invasive ventilation and put in a prone position shortly after admission, with a P/F ratio of 38. Amoxicillin with clavulanic acid 1.2 g IV three times a day and clarithromycin 500 mg IV twice daily were started in the emergency room and continued in the intensive care unit. Noradrenaline was started and escalated until 50 ug/min (1 mcg/kg/min). Lactate levels were then normalized. Transthoracic echocardiography showed a bicuspid aortic valve with mild aortic regurgitation and severe mitral regurgitation (Figure 3) with leaflet thickening and calcification (Figure 4) but without atrium enlargement.

**Figure 5:**
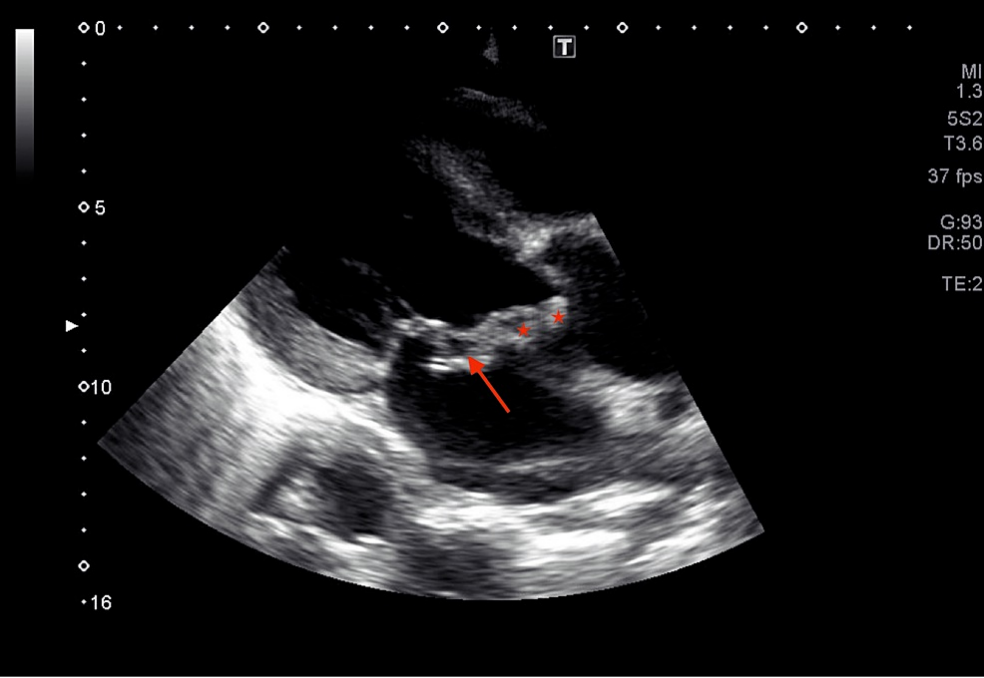
Transthoracic echocardiography showing endocarditis Transthoracic echocardiography in parasternal long-axis view showing mitral-aortic intervalvular fibrosa abscess (stars) with mitral valve abscess (arrow).

Once transesophageal echocardiography was performed and transthoracic echocardiography was reviewed, a mitro-aortic abscess was identified (Videos 1, 2) with a mitral-aortic intervalvular fibrosa abscess (Figure 5) and a perforated mitral leaflet, leading to severe mitral regurgitation (Video 2). Streptococcus mitis, with no antibiotic resistance, was isolated from the blood samples, and the antibiotic was changed to 18 million units of penicillin per day. Abdomen CT was performed with no signs of colonic polyps.

**Video 1:** Bicuspid aortic valve with abscess

**Video 2:** Mitral valve endocarditis Mitral valve endocarditis complicated by mitral valve abscess, which perforated, leading to severe mitral regurgitation.

Despite adequate medical treatment with resolution of shock and decrease in inflammatory markers, respiratory failure and weaning from the ventilator proved virtually impossible to manage due to pulmonary edema. He was transferred to the cardiac surgery department of a tertiary hospital, where they performed mitral valvuloplasty and closure of the valve perforation with an autologous pericardial patch. A bioprosthetic 27 mm aortic valve was put in place, and the patient was successfully weaned off the ventilator. Valve cultures were negative.

After initial improvement, he developed new signs of respiratory distress with increased needs for O_2_ levels and concurring new infiltrates in thoracic X-ray and CT scan. Transthoracic echocardiography showed globally reduced systolic heart function, with a minor mitral regurgitation. There was no sign of any other active infection, and blood and respiratory cultures were negative. Both cryptogenic organizing pneumonia and lung injury after cardiopulmonary bypass were thought, and he was started on methylprednisolone 1 g/d for three days. Over the next few days, the O_2_ levels were reduced, and the patient was transferred to the cardiology ward under 5 L/min of oxygen.

A colonoscopy was scheduled to be performed once the patient was discharged from the hospital to rule out gastrointestinal cancer.

## Discussion

Infective endocarditis alone is a life-threatening disease, and its complications (myocardial or paravalvular abscess, fistula, or pseudoaneurysms) can further aggravate its prognosis. Among the most frequent complications, valvular destruction is one of the most explored in clinical practice, with the search for new-onset heart murmurs. It can be present in up to 20% of patients who die of infective endocarditis and has an incidence of 10-20% [5,6].

Such clinical judgment is more challenging for a patient who is naïve to medical care. Still, the severe mitral regurgitation with a normal-sized left atrium was a significant clue for valvular destruction. Nevertheless, sometimes myocardial abscesses can present with thickening leaflets, which is not unexpected once a tendinous cord rupture is considered. This patient's transthoracic echocardiography was not specific for endocarditis, and the hypothesis of an acute mitral regurgitation due to a mitral cord rupture was even considered. However, once transesophageal echocardiography was performed, the abscesses were clear to see, and the transvalvular flow due to its rupture was noted. A myocardial abscess can be diagnosed when a nonhomogeneous mass or thickened area of the myocardium is seen. When the cardiac conduction system is involved, these abscesses can rupture, causing valvular leaks or new-onset heart block [6]. The presence of a cardiac abscess increases the mortality risk of a patient with infective endocarditis by 50% to 100% [7]. Our patient presented with an interesting case evolution, with a suspected first strike with bilateral pneumonia, followed by bacteremia with bicuspid aortic valve implantation and abscess formation. The mitral-aortic intervalvular fibrosa involvement and the perforated aortic valve abscess contributed to mitral valve endocarditis. In the end, with mitral valve perforation and major mitral regurgitation, pulmonary edema developed, contributing to hospital admission with a PaO_2_/FiO_2_ ratio of 38.

Viridans group streptococci usually have low pathogenicity in non-immunosuppressed patients. On the other hand, severe infections are more common in immunocompromised patients with gastrointestinal tract neoplasm or cardiac abnormalities, both in adults and in pediatric patients [8,9]. Patients with Streptococcus mitis bloodstream infection have a 19.4% risk of developing infective endocarditis and, in immunocompromised patients, has been shown as a possible agent for pneumonia with unexplained pulmonary infiltrate [9,10]. Our patient had no medical history that could categorize him as an immunocompromised patient, but gastrointestinal cancer was still to be excluded.

On the other side, patients with a bicuspid aortic valve have a 10-30% risk of developing infective endocarditis [11]. Such a condition affects 1-2% of the population, and its presence is an independent predictor factor for periannular complications in patients with infective endocarditis [11]. Both our patient's bicuspid aortic valve and poor dentition made him suitable for infective endocarditis, with significant valve complications and mitral-aortic intervalvular fibrosa abscess.

Our patient had never been in contact with the health care system. There were no medical records that could elucidate his previous heart history or lung diseases. His first blood gases showed a pO_2_ of 39 mmHg on 100% FiO_2_ on CPAP, with good tolerance, despite severe pneumonia and severe mitral regurgitation, which made us think he would have chronic hypoxia.

The risk of lung injury after cardiopulmonary bypass is well known, but most of its approach is centered on averting lung damage more than treating it [12]. The pathophysiology of this injury has different causes; one of them is lung ischemia and reperfusion injury caused by only maintaining the blood perfusion to the lungs through the bronchial arteries, which are typically responsible for only 3-5% of the total lung supply [13]. This, together with the systemic inflammatory response created by the extracorporeal circuit, can lead to lung injury. Since our patient presented with new-onset hypoxemia with bilateral and diffuse alveolar opacities and multifocal areas of airspace consolidation with preserved lung volumes, and nothing was supporting an infectious cause, corticosteroids were started. The corticosteroid used in our patient was methylprednisolone 1 g/d for three days, followed by tapering, considering the dosage recommended for cryptogenic pneumonia [14]. In the Dexamethasone for Cardiac Surgery (DECS) trial and Dexamethasone for Cardiac Surgery II (DECS-II) trial of perioperative use of corticosteroid therapy for enhancing patient recovery after cardiac surgery, the dosage of methylprednisolone was 500 mg [15].

Although this patient's respiratory failure was multifactorial, with ongoing factors contributing to it, the significant improvement noted after corticosteroids supported both these diagnoses.

## Conclusions

Streptococcus mitis bloodstream infection seems to be associated with an immunosuppressed state or cardiac abnormalities, like a bicuspid aortic valve, that predispose patients to the development of infective endocarditis. Its complications (myocardial or paravalvular abscess, fistula, or pseudoaneurysms) can further aggravate its prognosis. The formation and rupture of abscesses predispose these patients to major cardiac complications. Our patient had a bicuspid aortic valve with mild aortic regurgitation, severe acute mitral regurgitation, and mitral and aortic abscess, with the need for cardiac surgery. He was submitted to mitral valvuloplasty with closure of the valve perforation and placement of a bioprosthetic aortic valve. After surgery, despite cardiac improvement, surgical complications like lung injury can drastically aggravate a patient’s condition. This type of patient needs intensive care admission to wholly support their organ dysfunctions and optimize a multidisciplinary approach for such high mortality risk conditions.
